# Roast Meats

**Published:** 1886-01

**Authors:** 


**Roast Meats.**—To roast properly, meat should be placed a good distance from the fire and brought gradually nearer when about half done. If it should be basted frequently and when nearly done flowered to make it look frothed. The time required to roast a small fowl or chicken is twenty minutes ; for a good-sized turkey one hour to one hour and fifteen minutes ; for ten pounds of beef, two hours to two hours and thirty minutes ; for a large fowl, one hour and forty-five minutes ; for a neck of mutton, one hour and thirty minutes ; a goose, one hour ; to boil chicken, twenty minutes.

